# Key elements and contextual factors that influence successful implementation of large-system transformation initiatives in the New Zealand health system: a realist evaluation

**DOI:** 10.1186/s12913-023-10497-5

**Published:** 2024-01-10

**Authors:** Kanchan M Sharma, Peter B Jones, Jacqueline Cumming, Lesley Middleton

**Affiliations:** 1 Te Tai Ōhanga– The Treasury, 1 The Terrace, 6011 Wellington, New Zealand; 2 Department of Medicine, Faculty of Medical and Health Sciences, University of Auckland, 34 Princes Street, Auckland CBD, 1010 Auckland, New Zealand; 3 Health Services Research Centre, Faculty of Health, Victoria University of Wellington, Kelburn Parade, 6012 Kelburn, Wellington, New Zealand; 4 Faculty of Health, Victoria University of Wellington, Kelburn Parade, 6012 Kelburn, Wellington, New Zealand

**Keywords:** Complex adaptive system, Large-scale change, Health care reform, Realist evaluation, Continuous improvement, Collaborative networks

## Abstract

**Background:**

Despite three decades of policy initiatives to improve integration of health care, delivery of health care in New Zealand remains fragmented, and health inequities persist for Māori and other high priority populations. An evidence base is needed to increase the chances of success with implementation of large-system transformation (LST) initiatives in a complex adaptive system.

**Methods:**

This research aimed to identify key elements that support implementation of LST initiatives, and to investigate contextual factors that influence these initiatives. The realist logic of enquiry, nested within the macro framing of complex adaptive systems, formed the overall methodology for this research and involved five phases: theory gleaning from a local LST initiative, literature review, interviews, workshop, and online survey. NVivo software programme was used for thematic analysis of the interview, workshop, and the survey data. We identified key elements and explained variations in success (outcomes) by identifying mechanisms triggered by various contexts in which LST initiatives are implemented.

**Results:**

The research found that a set of 10 key elements need to be present in the New Zealand health system to increase chances of success with implementation of LST initiatives. These are: (i) an alliancing way of working; (ii) a commitment to te Tiriti o Waitangi; (iii) an understanding of equity; (iv) clinical leadership and involvement; (v) involved people, whānau, and community; (vi) intelligent commissioning; (vii) continuous improvement; (viii) integrated health information; (ix) analytic capability; and (x) dedicated resources and time. The research identified five contextual factors that influenced implementation of LST initiatives: a history of working together, distributed leadership from funders, the maturity of Alliances, capacity and capability for improvement, and a continuous improvement culture. The research found that the key mechanism of trust is built and nurtured over time through sharing of power by senior health leaders by practising distributed leadership, which then creates a positive history of working together and increases the maturity of Alliances.

**Discussion:**

Two authors (KMS and PBJ) led the development and implementation of the local LST initiative. This prior knowledge and experience provided a unique perspective to the research but also created a conflict of interest and introduced potential bias, these were managed through a wide range of data collection methods and informed consent from participants. The evidence-base for successful implementation of LST initiatives produced in this research contains knowledge and experience of senior system leaders who are often in charge of leading these initiatives. This evidence base enables decision makers to make sense of complex processes involved in the successful implementation of LST initiatives.

**Conclusions:**

Use of informal trust-based networks provided a critical platform for successful implementation of LST initiatives in the New Zealand health system. Maturity of these networks relies on building and sustaining high-trust relationships among the network members. The role of local and central agencies and the government is to provide the policy settings and conditions in which trust-based networks can flourish.

**Other:**

This study was approved by the Victoria University of Wellington Human Ethics Committee (Ethics Approval Number 27,356). The research was supported by the Victoria University of Wellington research grant (222,809) and from the University of Auckland Department of Medicine research fund (H10779).

**Supplementary Information:**

The online version contains supplementary material available at 10.1186/s12913-023-10497-5.

## Background

This research disentangles the unique contribution of different elements of a large-system transformation (LST) initiative to distil guiding principles for the developers and implementors of future initiatives.

For this research, we adopted the definition of LST initiatives provided by Best et al. [1]:

‘Interventions aimed at co-ordinated, system wide change affecting multiple organisations and care providers, with the goal of significant improvements in the efficiency of health care delivery, the quality of patient care, and population-level patient outcomes’ (p. 422).

LST initiatives are not about organisational changes or incremental improvements of current processes [1, 2]. LST initiatives are complex interventions implemented in a complex system.

Health systems are living and adaptive complex systems in which relationships, connections and interactions influence the behaviour of those who work in and use the system [3–5]. Organisational reforms and small-scale incremental improvements are not enough to improve the performance of these systems. LST initiatives that capitalise on key features of complex adaptive systems, such as use of informal networks and a deep understanding of how contextual factors influence LST initiatives, may be more likely to achieve the desired outcomes [1, 2, 6–8].

The initiative we investigated, known as the New Zealand (NZ) System Level Measures (SLM) programme, met three key aspects that have been theorised as characteristics of LST initiatives, as they (i) are broad and widespread across geographical boundaries, multiple organisations or across professional groupings; (ii) challenge current ways of thinking and seek paradigm shifts in mindsets, processes and relationships; and (iii) affect people and require co-ordination across multiple systems nested within a macro system [2].

The SLM programme was designed by health system leaders from the NZ Ministry of Health (Manatū Hauora), District Health Boards (DHBs) and Primary Health Organisations (PHOs) to enhance a collaborative way of working beyond organisational and professional boundaries, as well as to address health inequities and encourage continuous learning and quality improvement [9]. The SLM programme was implemented through Health Alliances. Alliances were informal networks in the NZ health system that were introduced to integrate the planning and delivery of health care between DHBs and PHOs [10].

The evidence base for successful implementation of LST initiatives stresses the importance of rich descriptions of what works for whom under what circumstances [1, 2, 6, 8, 11–13]. The focus is less about meeting performance targets and is more about iterative planning and practice cycles to shift system behaviour. Careful attention must be paid to relationships and connections that need to be thoughtfully crafted, nurtured and developed. For this research, this evidence base is referred to as the programme architecture that underpins efforts to successfully implement LST initiatives. This programme architecture is useful to bridge the gap between the theory of LST initiatives and the reality of implementing these initiatives in a complex adaptive system.

Our goal was to use the insights gained from the SLM programme and evidence from knowledge of those working in the health system to uncover the key elements that support successful implementation of LST initiatives. Further, we aimed to investigate and report on how contextual factors that NZ Alliances worked in influenced successful implementation of these initiatives, and to share these revelations with decision-makers in other regions and countries.

To achieve the research aims, the following two questions guided the research:

- What are the key elements that support successful implementation of LST initiatives in the NZ health system?
- What contexts and mechanisms influence NZ Alliances’ ability to successfully implement LST initiatives?

## Methods

### The NZ health system

The NZ health system is predominantly funded from general taxation. At the time of the research, Manatū Hauora had overall leadership of the health and disability system that included policymaking, management, and monitoring of health status, along with some purchasing/commissioning responsibilities (e.g., public health, well child, maternity, and ambulance services). Publicly financed health care was delivered through 20 DHBs, who oversaw health services in their districts. Most government health funding was distributed to DHBs using a weighted population-based funding formula [14]. DHBs delivered a large range of services themselves (e.g., hospital and hospital-related community services) and purchased/commissioned a range of services from for-profit and not-for-profit privately owned providers (e.g., primary healthcare, home care and residential rest home care).

PHOs are not-for-profit meso-layer organisations that were funded by DHBs, using a weighted capitation formula, to provide comprehensive primary care services through their member general practices. General practice teams provide the first point of contact in the health system and are gatekeepers to other primary care services (e.g., medicines, laboratory tests), secondary care and some community services.

Citizens choose the general practice they enrol with, and general practices choose the PHO they become members of [15]. PHOs pass on funding for first level services to their member general practices using the same weighted capitation formula that the DHBs use to fund PHOs. The funding arrangements in NZ means that general practices retain the right to charge co-payments to access primary care services. Fees are set by general practices with limited rules around frequency of increases and the maximum annual percentage increase as agreed with their contracting PHO and DHB [15]. The co-payment system remains in place today. A simplified visual description of the NZ health system before the 2021 reforms is shown in Supplementary Figure A.

Māori are the indigenous population of NZ. The British Crown and Māori rangatira (chiefs) signed te Tiriti o Waitangi (the Treaty of Waitangi) to live together under a common set of laws and agreements [16]. Under te Tiriti o Waitangi (te Tiriti) principles, Manatū Hauora, as a Crown agent, has responsibility to work together with iwi (Māori tribe), hapū (sub-tribe), whānau (family or extended family) and Māori to plan, develop, and deliver health and disability services to ensure Māori receive equitable health care and have equitable health outcomes as pākehā (New Zealanders of European descent) while protecting Māori cultural concepts, values and practices [16, 17].

The NZ Burden of Diseases study shows that New Zealanders’ health is improving, with recent increases in life and health expectancy [18]. However, NZ still has high health loss from coronary heart disease, chronic obstructive pulmonary disease, chronic kidney disease, bowel cancer, self-harm, and musculoskeletal disorders. Health inequities persist between genders, generations, and ethnic and socio-economic groups [18, 19]. Health conditions that are both preventable and amenable to timely medical interventions through equitable access to health services make a significant contribution to the lower life expectancy for Māori and Pacific populations. Nearly half of all premature deaths in Pacific (47.3%) and over half of all deaths in Māori (53%) have an avoidable cause compared to under a quarter of deaths (23.2%) among non-Māori non-Pacific populations [20].

The relationship between Māori and the Crown is one of continued negotiation. Any LST initiative needs to demonstrate compliance with te Tiriti, reduce health inequities and improve health outcomes for Māori.

There have been national policies and directions to promote changes and improvement in the system, with a desire for health services to be patient-centred, high quality, co-ordinated, integrated, and equitable. This has included the introduction of national initiatives such as the NZ Triple Aim [21], the requirement for DHBs and PHOs to form Alliances to enhance integration of hospital and primary care [22], and national strategies such as the NZ Health Strategy [23], the Primary Health Care Strategy [24], and He Korowai Oranga and Whakamaua (Māori Health Strategies) [17].

Despite three decades of policy initiatives to improve integration of health care, scholars and reviewers regularly conclude that the delivery of health care remains fragmented and focused on institutional arrangements [25–28]. The governance and institutional arrangements that separate service delivery of hospital, primary and community services, and the interests of powerful professional groups, have led to each service or group looking after their own interests and those of the patients in their service or speciality; and not the broader interests of patients and the population perspective [26, 29, 30].

The NZ health system has undergone a significant reform in 2022 that disestablished 20 DHBs and established two new national agencies: Te Whatu Ora - Health New Zealand and Te Aka Whai Ora - Māori Health Authority. Te Whatu Ora replaced DHBs and oversees the planning, funding, and delivery of public health services. Te Aka Whai Ora advises the government on all aspects of Māori health policy, monitors and reports on Māori health outcomes, partners with other organisations at all levels to integrate te Tiriti principles into policy, planning and service delivery, and strengthens the Māori workforce. Manatū Hauora remains the chief steward of the health system, responsible for policymaking and monitoring performance of the two new agencies. The intent of the reforms was to reduce variation in health care delivery and outcomes, increase efficiency and effectiveness by replacing 20 DHBs with a single national agency, and to ensure access to equitable health services and equitable health outcomes for Māori and other priority population groups [31].

### Approach and design

This research was informed by the realist logic of enquiry, nested within the macro framing of complex adaptive systems.

A complex adaptive system is an open system with blurred boundaries, which has a large number of agents who can simultaneously be members of several sub-systems, subject to change [3, 32]. In a complex adaptive system relationships, connections and interactions between system agents create feedback loops. These feedback loops influence the behaviours of agents in the system, causing them to learn, adapt and create further feedback loops. Future interactions cannot be predicted reliably from past interactions; history cannot be undone, but history influences present interactions [32, 33]. Outcomes from programmes or transformation initiatives in a complex adaptive system are non-linear, therefore a focus on interactions between sub-systems and the influence of contexts is required to understand how the system works.

Our research questions centred on gaining a deeper understanding of how the LST initiative worked in different contexts, so a realist research design, as developed by Pawson and Tilley [34], was chosen to further shape this research. The realist approach offers a framework to uncover the reasoning of health system agents and the influence of social and cultural conditions in which LST initiatives are implemented. We followed a logic of enquiry in which outcomes follow from mechanisms acting in the contexts of the system [34]. Following the tenets of this approach we adopted methods to test and refine programme theories to understand how mechanisms that operate according to context influence programme outcomes [34–37].

Mechanisms are underlying, unseen processes that exist in a system and influence outcomes depending on the circumstances or contexts. Mechanisms cannot be observed using methods to determine programme outcomes. They exist as part of a whole system, are triggered by contextual factors, are explanatory, have empirical content and are testable [38–40]. Mechanisms can be either positive or negative, it is necessary to understand both to create the environment for an intervention to produce the desired outcomes.

Contexts describe the organisational, social, cultural and political conditions that trigger the mechanisms, which then determine the programme outcomes [38, 40]. Each context in which a programme is implemented, for example using a top-down or a bottom-up approach for LST initiatives, triggers a different set of mechanisms which influences whether key outcomes (desired changes) are achieved. Context can be both an enabler and a barrier. A context that is enabling in one place can be a barrier in another owing to local needs and capability.

In our first research question we have used the term ‘element’ when we are referring to an elemental idea encompassed within the SLM programme. From our insights gained through implementation of the SLM programme, we knew there were multiple components that characterised success of the programme. For our research participants, we wanted a term that provided an accessible entry point to discuss these different components with practitioners. As Pawson has pointed out, realist models should be comprehensive enough to cover the many complexities within a programme, whilst still being readily intelligible to bring a sense of recognition to any practitioner [41]. In this research, the term element became the shorthand title for components that were contributing to the successful implementation of the SLM programme. Our attention then turned to explaining how different elements of the programme worked through CMO configurations. This differentiation echoes Dalkin et al. [42] work distinguishing between resources as components in the programme and the realist emphasis on how practitioners’ reason in response to that resource.

Mid-range theories in the form of context-mechanism-outcome (CMO) configurations explain what are the mechanisms that explain how and why reality unfolds as it does in a particular context or what works for whom under what circumstances and why [34]. Mid-range theories are essential for realist research [34]. They guide us to look for CMOs, in particular the mechanisms, as these are not directly observable [43]. In this research, CMOs offer a way to build on the lessons of the SLM programme and to test theories with a wide range of participants from across the health system (senior leaders to frontline staff).

### Data sources and analysis

The research was conducted between November 2018 and December 2019 and included five phases.

#### Phase 1– theory gleaning

The insights and knowledge from implementation of the SLM programme was used to create initial theories. This included identifying a list of key elements that supported the implementation of the SLM programme (listed in the first column of Table 1), and Initial Programme Theories (IPTs) on how these elements worked in different contexts to influence successful implementation (outlined in Table 2). For this research, successful implementation included a robust evidence-based planning process, full implementation of the agreed plan, and continuous reflective learning. The key elements and the IPTs were gained by documenting first-hand insights and knowledge of two authors (KMS and PBJ) involved in the development and implementation of the SLM programme and informal conversations with those involved in the implementation of the programme. During the three years of implementation, the two authors frequently attended Alliance Leadership Team meetings where they observed behaviours and listened to conversations that revealed the inner workings of these Alliances. The two authors also met with numerous clinical and operational leaders and frontline implementers of the programme. The observations and revelations acquired from these meetings together with the knowledge gained from the review of the SLM programme implementation plans and progress reports allowed the authors to identify the key elements and form the IPTs through abductive and retroductive reasonings.

**Table 1:** Consolidation of key elements from research phases

Insights from SLM programme	After literature review	After interviews	After workshop	After online survey
Alliancing way of working	Alliancing way of working	Alliancing way of working	Alliancing way of working	Alliancing way of working
Clinical leadership and engagement	Clinical leadership and engagement	Clinical leadership and involvement	Clinical leadership and engagement	Clinical leadership and involvement
Use of commissioning cycles	Use of commissioning cycles	Use of commissioning cycles	Intelligent commissioning	Intelligent commissioning
Continuous quality improvement focus	Continuous quality improvement focus	Continuous quality improvement focus	Continuous improvement	Continuous improvement
Information and communication technology	Integrated health information	Integrated health information	Integrated health information	Integrated health information
	Analytic capability (new element)	Analytic capability	Analytic capability	Analytic capability
Community and patient engagement	Engagement with patients and communities	Involved people and communities	Engaged people, whānau and community	Involved people, whānau and community
		Dedicated resources and time (New element)	Dedicated resources and time	Dedicated resources and time
			Commitment to te Tiriti o Waitangi (New element)	Commitment to te Tiriti o Waitangi
			Understanding of equity (New element)	Understanding of equity

**Table 2:** Initial Programme Theories for successful implementation of the SLM programme

Context	Mechanism	Outcome
Distributed leadership from DHBs	A commitment from Alliance members to implement the SLM programme	Full implementation of the agreed plan
A history of working together at the district level	High trust relationships among senior operational and clinical leaders in the district	A robust evidence-based planning process
Quality of relationships between DHBs and PHOs	High trust relationships among senior operational and clinical leaders in the district	A robust evidence-based planning process
Maturity of Alliances	An agreement to a shared vision and goals for the district	Full implementation of the agreed plan
Capacity and capability of Alliances	Recognition of the importance of investment in improvement capacity and capability	Continuous reflective learning
Continuous improvement culture at DHBs and PHOs	Active support from senior leaders at DHBs and PHOs for implementation of the SLM programme	Continuous reflective learning
Collaborative design of the programme with a continuous improvement approach and implementation through Alliances	Buy-in from the sector, in particular clinical leaders, and frontline health care professionals with the implementation of the SLM programme	A robust evidence-based planning process (and not seen as a compliance exercise)

The relationship cultivated by the two authors gave them access to Alliances and senior leaders that may not usually be available to a researcher. This relationship and access were crucial to form the IPTs and to recruit information-rich senior system leaders for the workshop (phase 4). The prior knowledge and experience also created a conflict of interest and introduced potential bias, for which mitigations are discussed further in the strengths, limitations, and implications section later.

#### Phase 2– literature review

An iterative review of published and grey literature was undertaken at all research phases. This enabled the research to be flexible and respond to emerging findings. First, a broad range of keywords were used to search published literature using keywords in the OVID and PUBMED databases relating to performance, governance, accountability, transformation and measuring quality improvement. The search was limited to English language from 2008 to 2018. Second, more keywords were used to search literature on systems and complexity theories and their application in health systems world-wide and LST initiatives in the health system. This search included using Google and visiting known quality improvement websites in the United States, United Kingdom, Canada, Australia, and NZ. Third, literature review was used to identify published LST initiatives in sectors other than health. Finally, a continuous snowball approach was used to identify further relevant published and grey literature. This included checking reference lists of previously identified literature, reviewing citation lists and reviewing newsletters from research institutes. The evidence from this phase was used to refine the list of key elements that support successful implementation of LST initiatives and to further identify contexts and mechanisms that influence it.

#### Phase 3– interviews

Purposeful sampling technique was used to recruit senior system leaders for interviews (n = 12). Participants were information rich, usually in charge of initiating and supporting the implementation of LST initiatives and making decisions relating to funding and resource allocations so could provide a view on leading LST initiatives in a complex system. Table 3 shows the profile of interview participants. The interview was designed and conducted using the guide published by Manzano [44]. Seven pre-determined questions ranging from semi-structured, structured prompts and open-ended conversations were used. Written consent was obtained for all interviews. For each element, participants were read out the description of the element and were then asked to rate the extent that they agreed or disagreed with the element being necessary to increase the chances of success with implementation of LST initiatives. Participants used a five-point Likert scale (strongly disagree, disagree, neutral, agree, and strongly agree) to rate the element. In some instances, participants chose to explain their rating, in others they did not. Once participants had finished rating the elements, they were asked to identify additional elements. The teacher-learner method was used to test and uncover CMO theories. We introduced the IPTs to participants and then sought their perspective. This allowed us to test our initial theories and uncover new ones. As interviews progressed, theories discovered from earlier interviews were also tested. NVivo software programme was used to aid thematic analysis of the interview transcripts. Transcripts of the interviews were coded deductively for key elements, and inductively to capture additional elements and CMO theories. Direct quotes from the transcripts were also used with all identifying information removed to protect participants’ anonymity.

**Table 3:** Interviewee profiles

Interviewee Profile
Health consultant and involved in the development of the SLM programme
Academic and health researcher in NZ
Health consultant and previous Chief Executive of a DHB
Health consultant and direct experience in the NZ health system
Māori clinician and involved in the development of the SLM programme
Clinician and leader of an LST initiative in Scotland
Health consultant and direct experience in the NZ health system and NHS
Health researcher of LST initiatives in complex adaptive systems
Clinician and leader of an LST initiative in Australia
Managing director– construction company in NZ
Technology and risk manager– NZ Bank
Strategic management consultant– NZ, Australia, and UK

#### Phase 4– workshop

The aim of the workshop was to use the knowledge of those working in the NZ health system to refine the key elements needed to support the successful implementation of LST initiatives and to identify the contexts and mechanisms that influence these initiatives. Participants (n = 10) were senior leaders and clinicians who were involved in the design or the implementation of the SLM programme, those who had significant experience and knowledge about the programme, and those charged with making major strategic decisions about where effort goes towards supporting these initiatives in their organisations. Other variables that were considered in the selection of the participants were: recognised leaders in the health system; Māori, equity, rural, nursing, and allied health perspectives; and advocates of service users who understood the complexity of the health system. Participation in the workshop was confirmed once written consent was received from the participant and their Chief Executive.

The workshop was facilitated by one of the authors (PBJ, the SLM programme clinical lead) who had led the co-development of the SLM programme and was a practising clinician who therefore had the relevant skills, subject matter expertise and credibility to elicit information from participants about what worked and what did not work with the implementation of the SLM programme.

The workshop contained three sessions to reach consensus on the key elements that support LST initiatives, to define and describe the outcomes for the key elements identified, and to test and uncover contexts and mechanisms that influenced the successful implementation of LST initiatives.

Data from the workshop was collected and analysed in real-time throughout the workshop. Data from previous sessions informed subsequent sessions of the workshop. Parts of the workshop were audio recorded with participants’ written consent. NVivo computer software programme was used to group key thematic groups of texts from the transcripts and coded deductively. Direct quotes from the transcripts were also used with all identifying information removed to protect participants’ anonymity.

#### Phase 5– online survey

The aim of the survey was to consolidate the key elements and further test our IPTs and theories that emerged from interviews and workshop with those involved in the implementation of the SLM programme in DHBs and PHOs. These participants had an inside knowledge and experience of what influenced the successful implementation of the SLM programme in their districts.

Fifty-one respondents from DHBs and PHOs participated in the online survey. They were made up of health care professionals (n = 8), those in management or leadership roles (n = 33), and others such as quality improvement leaders and analysts (n = 10).

The survey instrument included 10 key elements and refined CMO theories consolidated from the interviews and the workshop. Participants were asked their level of agreement on key elements and refined CMO theories using a five-point Likert scale (strongly disagree, disagree, neither disagree nor agree, agree, and strongly agree). Using open-ended questions, participants were then asked to identify further contexts and mechanisms that influenced the implementation of the SLM programme in their district.

NVivo computer software programme was used for thematic analysis of the survey data. Direct quotes from the survey were also used with all identifying information removed to protect participants’ anonymity.

## Results

### Key elements that support successful implementation of LST initiatives

The research identified 10 key elements that are needed to increase the chances of success with implementation of LST initiatives in the NZ health system. Table 1 shows the consolidation of key elements from the five research phases. Table 4 defines the final 10 key elements using evidence from literature and knowledge of research participants.

**Table 4:** Definition of key elements

Key element	Definition
Alliancing way of working	A clinically, community and iwi-led forum that brings all agents in the health system together with the aim of transforming services with people and equity at the centre of their decision-making [10, 45, 46].
Clinical leadership and involvement	Health care professionals providing leadership and system oversight with a focus on continuous quality improvement to create an environment for evidence-based clinical practice and team-based approaches to care delivery [47].
Intelligent commissioning	The process of continuously developing services and committing resources to enable the best health outcomes and wellbeing and that includes many activities ranging from health needs assessment, cultural paradigms, and development of pathways to service specification and contract management or procurement, underpinned by continuous improvement.
Continuous improvement	Systematic and sustained use of continuous quality improvement methods, measurement tools and feedback loops that provide opportunities for learning and build accountability in the system.
Integrated health information	Availability of technology, and health and social information, both identifiable and at population aggregate level, across the different parts of the system at local and national level. This readily accessible health information is responsive to needs and guides commissioning decisions.
Analytic capability	Ability to analyse, link clinical and administrative data, and produce insights and evidence for frontline staff to measure, understand and feedback data on clinical variation and outcomes.
Involved people, whānau and community	An approach that actively involves individuals, carers, hapū, whānau, iwi and communities in the design and delivery of health care to generate significant benefits to the health care and wellbeing of all people.
Dedicated resources and time	Appropriate continuous resourcing such as health workforce, funding, knowledge, time, project management support, administration support and the time and space to be successful.
Commitment to te Tiriti o Waitangi	Complying with principles of te Tiriti through equity and active protection to achieve equitable health outcomes for Māori; culturally appropriate health care that recognises and supports Māori models of care, and working in partnership with Māori in the governance, design, delivery and monitoring of health services [48].
Understanding of equity	Recognising different people with different levels of advantage require different approaches and resources to get equitable health outcomes [49]. Emphasis is given to Pacific and other high priority populations such as those with mental health conditions and with disability.

### Contexts and mechanisms that influence successful implementation of LST initiatives

#### Initial Programme theories (IPTs)

From engagement with those implementing the SLM programme (including insights, knowledge, and hunches of two authors KMS and PBJ), our initial theories of what made the programme work are outline in Table 2.

Insights from implementation of the SLM programme showed that NZ Alliances varied in form and function. In districts where relationships between a DHB and its system partners (in particular PHOs) were fraught, Alliances were perceived as bureaucratic networks that lacked mandate and resources, had low-trust relationships, and were ineffective in delivering any benefits for the local system. This led to variations in how successful districts were with the implementation of the SLM programme.

We tested and refined these theories using literature review, interviews, workshops, and an online survey. We used the realist framing of CMO with C being the contextual factors in which Alliances operate in, M being the underlying reasoning of health system agents involved in the implementation of LST initiatives, and O being the variations in the intermediate outcomes for successful implementation of LST initiatives.

While most of the IPTs were confirmed by research participants, evidence from knowledge of those working in the system and leading LST initiatives provided rich descriptions of mechanisms and outcomes that were triggered by the local and national contexts, examples of which are provided in Table 5.

**Table 5:** Examples of descriptions from participants

Participant	Direct quotes	Mapped to relevant CMO
Workshop participant	*“Sort of you’re always working against the history or you’re working with the history, one of the two.”*	A history of working together
Interviewee	*“I used to call it folklore– in NZ, clinicians and boards could recount stories, which were about creating mistrust and dwelling on grievances of the past. Trust was enhanced when previous efforts were successful and eroded when past grievances were embedded in folklore.”*	A history of working together Nurtured and sustained trust
Interviewee	*“We think that a great deal of the change that we need to make in the health system is dependent on having really strong collaborative relationships within and across organisations.”*	Quality of relationships Maturity of Alliances
Interviewee	*“So, it’s about learning to trust each other and to work in harmonious collaboration.”*	Maturity of Alliances Nurtured and sustained trust
Survey participant	*“Building trusted equitable relationships across the local health environment and using a collective impact approach with participants so that all parties have equal power at the decision-making table, rather than the party with the funding.”*	Quality of relationships Nurtured and sustained trust Distributed leadership from DHBs
Workshop participant	*“The DHB has to trust. It can’t sit there and say, well, I will trust you when you behave in a trustworthy way. It actually has to say, okay, we’re going to trust the system, and then work with it. That one action will change a whole heap of dynamics.”*	Nurtured and sustained trust Distributed leadership from DHBs
Interviewee	*“…working beyond and throwing (away) the boundaries of the organisation that you work for and are employed by, such as a DHB or a PHO when going into the room or throwing away the boundaries of the particular area of clinical practice that you happen to hail from and going into to work in the Alliance with the whole of system approach, essentially.”*	Maturity of Alliances

#### Final CMO configurations

Findings from this research identified six key contexts (five local and one national) that influenced successful implementation of LST initiatives, these are summarised in Table 6.

**Table 6:** Final key CMO configurations for successful implementation of LST initiatives

Context	Mechanism	Outcome
Distributed leadership from DHBs	Alliance members see sharing of power and feel less threatened	Sustained trust and commitment to an alliancing way of working
A positive history of working together	Presence of trust among senior leaders of health system partners	DHB senior leaders practise distributed leadership
Maturity of Alliances	Alliance members are able to navigate through disagreements, emerging issues, and changes in Alliance membership, and stay focused towards the shared vision	Alliance is cohesive and resilient and is able to successfully implement LST initiatives
Capacity and capability for improvement	System leaders recognise the importance of capacity and capability for implementation of LST initiatives	System leaders invest in organisational capacity and capability
A continuous improvement culture	Active support from senior system leaders for a continuous improvement approach	Organisations embrace critique and look to continuously improve their performance
Collaborative approach to design and implementation of LST initiatives (national context)	Confidence in system actors to be innovative and do things differently	Sustained engagement with implementation of LST initiatives

**Fig. 1:**
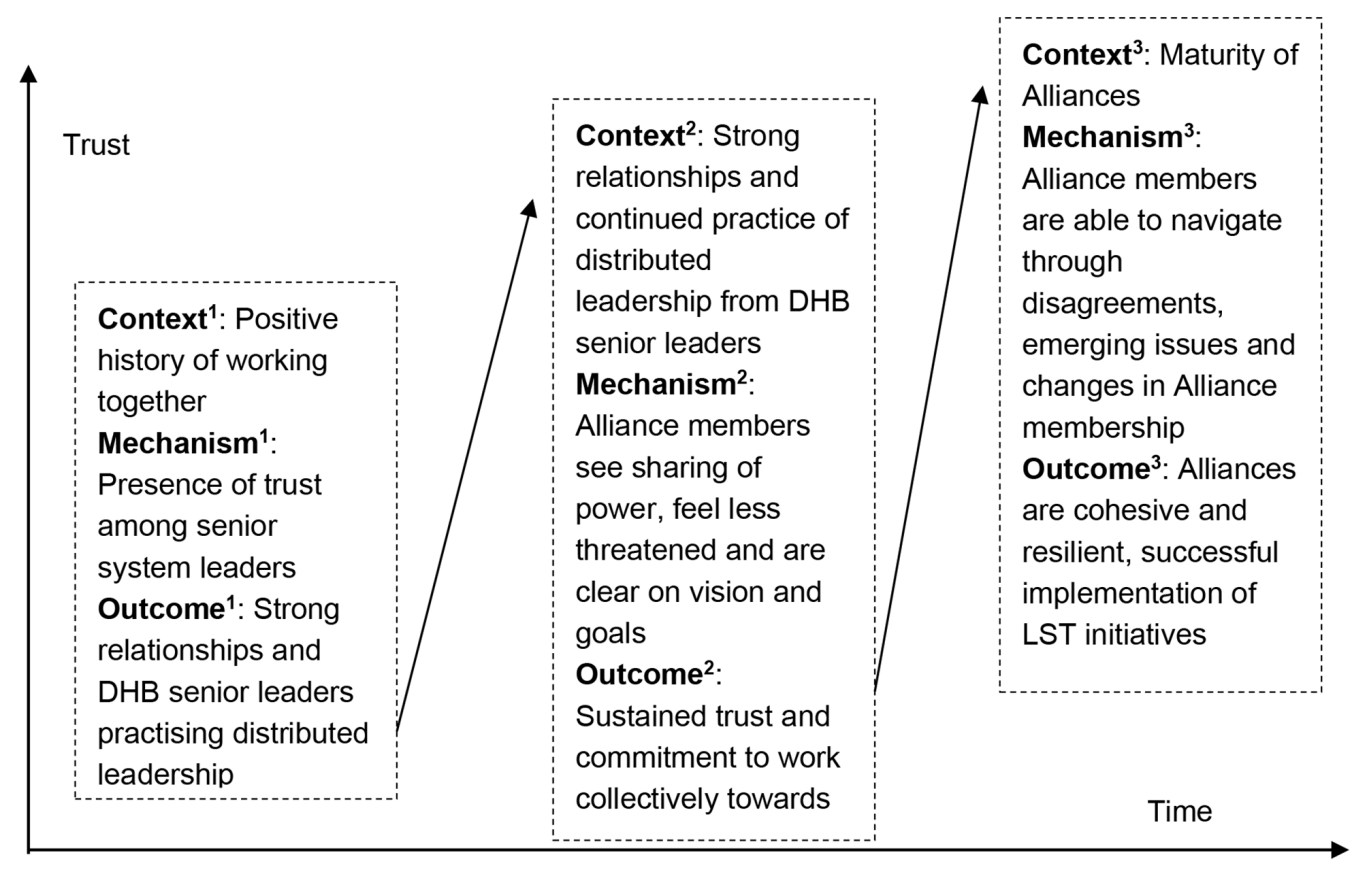
CMO interplay showing the ripple effect of the key mechanism of trust over time, adapted from Jagosh et al. [50]

A major finding of the study was that the first three contexts were inter-related and had a ripple effect on building and maintaining the key mechanism of trust. Figure 1, adapted from Jagosh et al. [50], illustrates this ripple effect.

The presence of trust among senior system leaders (M) triggered by a positive history of working together (C) led to strong relationships and DHB senior leaders practising distributed leadership (O). The strong relationships and power sharing through distributed leadership from DHB senior leaders (C) nurtured and sustained trust between senior system leaders (M) and led to these leaders agreeing a shared vision and goals for their local system and a commitment to work towards these through an alliancing way of working (O). The agreement and commitment among senior leaders were enabling contextual factors that increased maturity of Alliances (C). Mature Alliances (C) were able to navigate through disagreements, emerging issues, and changes in their membership (M), which led to them being cohesive and resilient and provided a critical platform to successfully implement LST initiatives (O). The key mechanism of trust, built and nurtured over time, increases maturity of informal networks, which then increases the chances of success with implementation of LST initiatives.

An important national context identified in the research was a collaborative approach to the design and implementation of LST initiatives, which enabled a bottom-up approach to policy development and gave system actors confidence to be innovative and do things differently. A collaborative approach ensures sustained engagement and buy-in for change efforts.

Using the realist analysis method adapted from Willis et al. [51], the local and national contexts, the factors that enable and constrain these, the mechanisms triggered and the outcomes that follow are described in the Supplementary Table A.

## Discussion

Findings of this research are congruent with evidence in literature [1, 52–54] that the desired health system transformation to deliver equitable health outcomes is more likely to be achieved through inter-organisational collaboration rather than competitive behaviours. For NZ, te Tiriti [48] and the Pae Ora (Healthy Futures) Act [31] requires that Māori are involved in the design and delivery of health care that meet the needs and aspirations of Māori.

Although the 10 key elements were not weighted in our analysis, feedback from participants indicated that the alliancing way of working is the key element that provided a critical platform for successful implementation of LST initiatives in the NZ health system. The presence of the remaining nine elements contribute to successfully implementing change at an organisational or a service level but do not on their own lead to successful implementation of LST initiatives that involve many organisations and agents present in a complex system.

Further, contexts such as a history of working together, the leadership style of system senior leaders, and the maturity of Alliances have a more profound influence on their success rather than a national policy direction. This is a common finding from the implementation literature: that ideas for change rarely filter through a complex adaptive system without being adapted and re-worked by those on the frontline [55] and that contextual factors, such as history of working together and leadership style, heavily influence the performance of networks such as Alliances [52].

The recent health reform has shifted away from Alliances to a locality approach. Localities are local networks comprising of local health service providers, social sector agencies, non-government organisations, iwi, local authorities, and consumers and communities. Te Whatu Ora has a legislative responsibility to ensure all of NZ is covered by a locality, that there is a plan outlining priority outcomes and services for the locality, and that Te Aka Whai Ora, iwi and Māori are involved in the development, implementation and review of the locality plan [31].

While a requirement to form integrated networks, such as Alliances or localities, can be mandated from the centre, the success of these networks relies on a positive history of working together in a high-trust environment. Change in health systems requires the buy-in, involvement and leadership of operational and clinical leaders across the health system. During the implementation of the SLM programme, buy-in was demonstrated by clinical leaders and staff prioritising the time to engage, their willingness for continuous learning to improve the quality of the SLM plans, their advocacy of the programme to their peers, managers, the Minister of Health and senior leaders in Manatū Hauora, and their perseverance with working in a collaborative way even when local relationships were fraught. Clinical leaders usually do not hold formal leadership roles and are traditionally siloed in their area of speciality or profession but are critical to the success of any change effort. The use of trust-based networks provides the platform for senior health system leaders (who often hold power) to share power with clinical, community, and other system agents. The sharing of power empowers and mobilises these agents in the system to think and act beyond their self-interest, profession, or organisation.

The NZ health system does not lack plans or policy initiatives to guide the improvement of system performance– long-term strategic plans, such as the NZ Health Strategy, Primary Health Care Strategy and He Korowai Oranga have existed since the early 2000s, as well as recently developed plans and legislation, such as Whakamaua and the Pae Ora Act. Policy initiatives such as the establishment of Alliances, have attempted to coalesce multiple health system agents to work beyond their organisational and professional boundaries in the best interests of patients and the populations they serve. The gap has been a lack of understanding of the unique contribution of different elements and the influence of contextual factors on design and implementation of LST initiatives. The focus needs to be on building health system leadership capacity and capability through understanding of the programme architecture that underpins efforts to successfully implement LST initiatives.

Appointments to key leadership roles and leadership development programmes need to focus on strong interpersonal competence such as, ability to build and nurture high-trust relationships, and not solely focus on individual skills, experience, and seniority in the system. Leaders who have ascended to their roles by working in the system have individual attributes and skills but may not necessarily understand the architecture that underpins successful implementation of LST initiatives or possess the social capital in the form of relational capability and behaviours needed to lead and sustain these initiatives.

It is a common phenomenon in a complex adaptive system for changes in key leadership roles to undermine system leadership capacity and disrupt the direction of travel for the system [56]. A collective leadership model provides stability and resilience to changes in senior leadership roles [57]. Collective leadership requires a platform for system actors to share knowledge and learn from each other. Informal trust-based networks provide the platform for collective leadership to build and sustain system leadership.

Research participants and the literature [53, 54, 58–62] are both strong on the need for dedicated resources for LST initiatives, both in the form of new resources and re-alignment or re-prioritisation and upskilling of current resources to match needs. In addition to an appropriate and sustainable health workforce, other resources needed for successful implementation of LST initiatives include project or programme managers, information technology capability, change management leadership and evaluation skills, and enough time for change to occur [58]. Senior health system and political leaders often underestimate the importance of these resources. These are often perceived as administrative functions, which are nice to have but not essential for a high performing health system. This perception needs to change for the health system to be successful with implementation of LST initiatives that will achieve the desired system transformation.

### Strengths, limitations, and implications

The prior knowledge of the two authors (KMS and PBJ) and their direct experience with leading the development and implementation of the SLM programme provided a unique perspective to the research. In realist research, prior knowledge of the research subject matter and purposeful selection of participants that are information rich are essential tenets, it allows the researcher to use personal experience and hunches to identify and test theories from within the system rather than looking in [34, 44]. However, our knowledge and experience also created a conflict of interest and introduced potential bias to the research. The conflict of interest was robustly considered during the ethics approval process and was managed in several ways such as, the informed consent process with participants. The bias was managed by selecting a wide range of data collection methods that allowed us to involve participants from different levels ranging from senior operational and clinical leaders to frontline staff, which enabled us to actively search for rival theories and build rigour to our theories. A key strength of this research was the involvement of senior health system leaders from NZ and overseas who did not hesitate to share their opinions, or challenge or disagree with our theories. The research also sought an outside perspective from those with knowledge and experience of leading implementation of LST initiatives in non-health industries to determine if there were elements and contextual factors that were common across complex systems. Another strength of this research was recognising both equity and te Tiriti o Waitangi in the programme architecture, which are important contexts for the modern-day NZ health system that aspires to meet the needs of a multi-cultural society while understanding and addressing biculturalism (Māori and Pākehā) that comes from te Tiriti.

The research findings provide a rich description of the programme architecture that underpins efforts to successfully implement LST initiatives in the NZ health system. These findings provide valuable insights and lessons to decision makers in other regions and countries and the CMO theories reported in this research add to the health system transformation literature, which is still considered young and scarce.

## Conclusions

Over last two decades, recognition that health systems are complex adaptive systems has challenged the traditional transactional paradigm. LST initiatives that capitalise on key features of complex adaptive systems are theorised to achieve the desired shift most health systems are seeking, to deliver health care that addresses health inequities and is people-centred and holistic.

This research affirms the use of informal trust-based networks to bring together many health system agents to deliver integrated people-centred health care. Performance of these networks cannot be mandated from the centre. It is underpinned by unprecedented levels of trust built and sustained through power sharing and commitment among partners to work towards a shared vision to overcome great challenges in a complex system in which no single player is likely to achieve individually. The role of local and central agencies and the government is to provide the policy settings and conditions in which trust-based networks can flourish. The research found that understanding of the programme architecture by health system leaders is critical. System leaders create the pre-conditions and influence the contexts for LST initiatives to be successfully implemented.

### Electronic supplementary material

Below is the link to the electronic supplementary material.

**Additional File 1: Table A:** Consolidated context-mechanism-outcome theories

**Additional File 2: Figure A:** Simplified visual description of New Zealand health system before the 2021 reforms

**Additional File 3:** Interview design and implementation

## Data Availability

Data sharing is not applicable to this article as no datasets were generated or analysed during the current study. All recordings and transcripts have been destroyed as per the research confidentiality agreement.

## Abbreviations

CMO: context-mechanism-outcome
DHBs: District Health Boards
LST initiatives: Large-system transformation initiatives
NZ: New Zealand
PHOs: Primary Health Organisations
SLM programme: System Level Measures programme
Hapū: Māori sub-tribe
He Korowai Oranga: Māori Health Strategy
Iwi: Māori tribe
Manatū Hauora: Ministry of Health
Māori: indigenous people of New Zealand
Pae ora: Healthy futures
Pākehā: New Zealanders of European descent or non-Māori
Te Aka Whai Ora: Māori Health Authority
Te reo Māori: Eastern Polynesian language spoken by the Māori people
Te Tiriti o Waitangi: The Treaty of Waitangi
Te Whatu Ora: Health New Zealand-
Whakamaua: Māori Health Action Plan
Whānau: family or extended family
