# Supplementary material for: Key elements and contextual factors that influence successful implementation of large-system transformation initiatives in the New Zealand health system: a realist evaluation
Source: BMC Health Serv Res. 2024 Jan 10;24:54. doi: 10.1186/s12913-023-10497-5 (PMC10782523; doi:10.1186/s12913-023-10497-5)
Supplement: Supplementary file 1 — Additional File 1: Table A: Consolidated context-mechanism-outcome theories [file 12913_2023_10497_MOESM1_ESM.docx]

**Table A - Consolidated context-mechanism-outcome theories for successful implementation of LST initiatives in the New Zealand health system**

| Context | Realist mechanism | *Constraining factors* | *Enabling factors* | Outcome |
| --- | --- | --- | --- | --- |
| History of working together and quality of relationships | Successful with previous change efforts  Presence of trust among senior leaders of health system partners  'Bottom-up' approach to change | Negative history of working together  ‘Master-servant’ dynamic between senior leaders in DHBs and PHOs  Power and control by senior DHB and PHO leaders  Focus on hospital  Top-down approaches to LST initiatives  Frontline health care professionals are not involved in design of solutions or their solutions are not implemented by operational leaders | Positive history of working together  Senior leaders value relationship and collective action through ‘bottom-up’ approaches to LST initiatives  DHB shares power with other system leaders  DHBs recognise the system outside the hospital  Frontline health care professionals are given tools, are involved in design of solutions and see their solutions being implemented | Enhanced trust  Enhanced quality of relationships  DHB senior leaders practise distributed leadership  Clinical and operational leaders are more willing to come together, share resources, data and ideas and attempt on change efforts  Clinicians feel empowered to improve the system they work in |
| Distributed leadership from DHB leaders | Alliance members see sharing of power and feel less threatened by the size of organisation or budget  Fosters a collective approach to implementation of LST initiatives  Staff are clear on vision and goals and use these to prioritise their work and feel confident to do things differently | Low-trust relationships between DHBs and PHOs  Command and control leadership style of senior leaders in DHBs and PHOs  Power and control by senior DHB and PHO leaders  Poor history of working together  Focus on hospital  Change fatigue | High-trust relationships between DHBs and PHOs  Collaborative, collective or distributed leadership styles of senior leaders in DHBs and PHOs  Positive history of working together  Strong and courageous leadership  DHBs recognise the system outside the hospital | Trust between senior system leaders is nurtured and sustained and there is a commitment to an alliancing way of working  Senior leaders in the district agree on an agreed integrated work programme for their district that includes a shared vision and goals; and all actors commit to working towards the agreed work programme |
| Maturity of Alliances | Alliance members are able to navigate through disagreements, deal with emerging issues, overcome changes in senior leadership roles and Alliance membership, and stay focused towards the shared vision | Lack of an Alliance  Lack of a whole-of-system approach  Lack of a shared vision and goals  Lack of resources to support the Alliance  Lack of sharing data and intelligence  Agreed Alliance charter in place but behaviours of members do not reflect the way of working described in the charter | High-trust relationships  Shared vision and goals  Commitment among Alliance partners to work towards shared vision and goals  Members of the Alliance live the agreed charter  Alliance has independent chair.  Alliance has mandate and embedded within DHB governance structure  Alliance is supported by lower-level structures such as working groups | Alliance is cohesive and resilient and is able to successfully implement LST initiatives |
| Capacity and capability | System leaders recognise the importance of capacity and capability for implementation of LST initiatives  Use of data and evidence in change efforts  Health care professionals see value in the use of integrated health information | Lack of awareness by senior leaders on the capacity and capability of their organisation  Power and control by senior DHB and PHO leaders  Frequent changes to senior leadership roles  Poor relationships  Lack of dedicated analytic resources, skilled project or programme managers, budget and evaluative processes  Lack of or out of date appropriate information technology tools  Clinicians not reimbursed for their loss of income or costs to attend meetings  Change management added to existing work demands of those in the system | Continuity of system leadership roles  Senior leaders value relationships  High-trust relationships  Willingness to share power, successes and failures Integrated health information  Analytic capability  Dedicated personnel with right skills recruited or re-directed to co-ordinate and or assist with change efforts  Time and incentives available for clinical leaders to engage in Alliance leadership team  Meetings held at times suitable for clinicians and frontline staff to attend  Clinicians provided with locum to backfill  Clinicians reimbursed for their loss of income and costs to attend meetings | System leaders invest in organisational capacity and capability  Willingness of system leaders to partner with other organisations to boost capacity and capability  Stronger clinical engagement in identification of meaningful quality improvement activities  Health care professionals more likely to share patient level data across the system |
| Continuous improvement culture | Active support from senior system leaders for a continuous improvement approach  Staff feel encouraged and supported to challenge status quo and try new initiatives | Sole focus on accountability reporting  Lack of organisational values or values do not support continuous improvement  Lack of awareness, availability and use of improvement science tools and methodology  Lack of buy-in from senior system leaders  Culture of blame | Balance between accountability and continuous improvement  Organisational values support continuous improvement  Use of constant feedback loops and plan-do-study-act processes  Use of evaluation processes  A permissive culture to try new initiatives (i.e., Safe to fail) | Organisations embrace critique and look to continuously improve their performance |
| Collaborative approach to design and implementation of LST initiatives | Confidence in system actors to be innovative and do things differently | Lack of dedicated resources and time  Lack of political mandate and credibility  Lack of financial incentives  Disconnect between national policies and planning processes | Dedicated resources and time  National improvement or seed funding available  Collaborative development and implementation of the LST initiatives  Financial incentives | Sustained engagement with implementation of LST initiatives |
