# Supplementary material for: Key elements and contextual factors that influence successful implementation of large-system transformation initiatives in the New Zealand health system: a realist evaluation
Source: BMC Health Serv Res. 2024 Jan 10;24:54. doi: 10.1186/s12913-023-10497-5 (PMC10782523; doi:10.1186/s12913-023-10497-5)
Supplement: Supplementary file 2 — Additional File 2: Figure A: Simplified visual description of New Zealand health system before the 2021 reforms [file 12913_2023_10497_MOESM2_ESM.docx]

Figure A: Simplified New Zealand Health System Structure – Before 2021 reforms

Co-payments

Central Government

Minister of Health

Ministry of Health

- Policy and regulation
- DHB funding and performance management
- National services
- Health workforce

20 District Health Boards (DHBs)

DHB provider arms

Hospital and hospital-related community services, public health services

Primary Health Organisations and General Practices

Other community, home, and residential care providers

Health and disability support service users
