# Supplementary material for: Key elements and contextual factors that influence successful implementation of large-system transformation initiatives in the New Zealand health system: a realist evaluation
Source: BMC Health Serv Res. 2024 Jan 10;24:54. doi: 10.1186/s12913-023-10497-5 (PMC10782523; doi:10.1186/s12913-023-10497-5)
Supplement: Supplementary file 3 — Additional File 3: Interview design and implementation [file 12913_2023_10497_MOESM3_ESM.docx]

Interview design and implementation

|  | Question | Realist logic |
| --- | --- | --- |
|  | Can you tell me little bit about your area of expertise? | Rapport building – to get them talking |
|  | *My understanding of complex adaptive systems is that these are open systems with blurred boundaries and unpredicted ways of workings, whose agents learn, interact and adapt their behaviours to a changing environment.*  Can you tell me your understanding of complex adaptive systems and how it relates to the (health system or your sector)? | Introduce the concept of systems thinking as a teacher  Switch to learner |
|  | *Literature shows that in order to effect change in a complex adaptive system, LST initiatives are required. By LST initiatives I am referring to co-ordinated, system-wide change involving multiple organisations that takes into consideration the relationships, interactions and behaviours of those in the system.*  To what extent do you agree or disagree with this?   \| Strongly disagree \| Disagree \| Neutral \| Agree \| Strongly Agree \| \| --- \| --- \| --- \| --- \| --- \| | Introduce large-system transformation (LST) initiatives as a teacher  Switch to learner and use prompt guide to refine theory |
|  | Insights from the System Level Measures programme and evidence from literature tells me the following elements are necessary to implement LST initiatives successfully and sustainably.  To what extend do you agree or disagree with each of these?   - 1. Alliancing way of working   *Alliancing is a clinically led, community engaged forum that brings different component parts of the health system together with the aim of transforming services so that they are best for the patient and best for the system. Alliancing is a way of working, reliant on trusted relationships and supported by enablers critical to continuous improvement. Successful Alliances bring alignment to multiple organisations and services present in a complex system, to achieve a common set of patient-centred goals. Alliancing is not about managing finances or organisational or professional accountabilities.*   \| Strongly disagree \| Disagree \| Neutral \| Agree \| Strongly Agree \| \| --- \| --- \| --- \| --- \| --- \|  - 1. Clinical leadership and engagement  \| Strongly disagree \| Disagree \| Neutral \| Agree \| Strongly Agree \| \| --- \| --- \| --- \| --- \| --- \|  - 1. Use of commissioning cycle   *Commissioning is the process of continuously developing services and committing resources to enable the best health outcomes to be achieved for individuals and the population, equity to be assured and experience enhanced within the resources available (Productivity Commission NZ).*   \| Strongly disagree \| Disagree \| Neutral \| Agree \| Strongly Agree \| \| --- \| --- \| --- \| --- \| --- \|  - 1. Integrated health information  \| Strongly disagree \| Disagree \| Neutral \| Agree \| Strongly Agree \| \| --- \| --- \| --- \| --- \| --- \|  - 1. Analytic capability  \| Strongly disagree \| Disagree \| Neutral \| Agree \| Strongly Agree \| \| --- \| --- \| --- \| --- \| --- \|  - 1. Continuous quality improvement focus  \| Strongly disagree \| Disagree \| Neutral \| Agree \| Strongly Agree \| \| --- \| --- \| --- \| --- \| --- \|  - 1. Engagement with patients and communities  \| Strongly disagree \| Disagree \| Neutral \| Agree \| Strongly Agree \| \| --- \| --- \| --- \| --- \| --- \|   Do you have anything else to add to this list? | Identify key elements. Start as a teacher outlining each theory and then switch to learner and use prompt guide to confirm or refute each theory. At the end look for additional information which may refine theory or introduce new ones. |
|  | *For the next set of questions, I would like to explore contextual factors that influence the key elements and what impact these have.*  *Let’s choose your top three key elements from the list above.*  What local circumstances, at the organisational level, influence these elements and in what way? What causes these effects? Give me an example | Exploring organisational contexts, mechanisms and outcomes  Conversational technique – switch between teacher and learner during the conversation depending on the interviewee engagement.  May test theories discovered in earlier research phases |
|  | What national, social or political circumstances influence your top three key elements and in what way? What causes these effects? Give me an example | Exploring national contexts, mechanisms and outcomes  Conversational technique – switch between teacher and learner during the conversation depending on the interviewee engagement.  May test theories discovered in earlier research phases |
|  | Is there anything else you can tell me on how to build capacity and capability for improvement that will enable successful and sustainable implementation of LST initiatives in the health system? | Concluding question  End the interview as a learner |
